# Recruitment strategies in a prospective longitudinal family study on parents with obesity and their toddlers

**DOI:** 10.1186/s12889-017-4038-9

**Published:** 2017-02-01

**Authors:** Sarah Bergmann, Anja Keitel-Korndörfer, Katharina Herfurth-Majstorovic, Verena Wendt, Annette M. Klein, Kai von Klitzing, Matthias Grube

**Affiliations:** 1 Integrated Research and Treatment Center (IFB) AdiposityDiseases, University of Leipzig, Philipp-Rosenthal-Strasse 27, 04103 Leipzig, Germany; 2 Department of Child and Adolescent Psychiatry, Psychotherapy, and Psychosomatics, University of Leipzig, Liebigstrasse 20a, 04103 Leipzig, Germany

**Keywords:** Family study, Recruitment, Cost effectiveness, Obesity, Corporate design, Child, Toddler

## Abstract

**Background:**

Recruitment of participants with obesity is a real challenge. To reduce time and costs in similar projects, we investigated various recruiting strategies used in a longitudinal family study with respect to their enrolment yield and cost effectiveness. Results may help other research groups to optimize their recruitment strategies.

**Methods:**

We applied different recruitment strategies to acquire families with children aged 6 to 47 months and at least one parent with obesity (risk group) or two parents of normal weight (control group) for a longitudinal non-interventional study. Based on four main strategies-via media, kindergartens, health professionals and focusing on the community-we examined 15 different subcategories of strategies. Based on enrolment yield and relative costs (e.g., material expenses, staff time) we analyzed the effectiveness of each recruitment strategy.

**Results:**

Following different recruitment approaches, 685 families contacted us; 26% (*n* = 178) of these met the inclusion criteria. Of the four main strategies, the community-focused strategy was the most successful one (accounting for 36.5% of the sample) followed by contacts with kindergartens (accounting for 28.1% of the sample). Of the subcategories, two strategies were outstanding: Posters (community-focused strategies), and recruitment via kindergartens using phone contacts rather than emailing. Only a small number of participants were recruited via announcements in newspapers (lower cost strategy), advertisements on public transport or face-to-face recruitment at various places (higher cost strategies).

**Conclusions:**

Results revealed that only a combination of different active and passive methods and approaches led to a sufficient sample size. In this study, recruitment via posters and contacting kindergartens on the phone produced the highest numbers of participants (high enrolment yield) at moderate costs.

## Background

Recruiting participants for clinical trials is a challenge [1]. Recruitment is often time-consuming, costly and a “learning by doing procedure” [2, 3], and this clearly applies to studies that include the whole family. Prior research has identified various barriers to recruiting families for clinical trials on obesity [4, 5]. One crucial impediment was parents’ denial or lack of awareness of children’s weight problems [6, 7]. Furthermore, weight problems are associated with shame [8]. People with obesity may tend to avoid such studies. On top of that, professionals who were supposed to identify weight problems in the population often failed to broach the issue of excess weight [4]. Finne and colleagues [4] further speculated that a lower treatment motivation in families with overweight children could be responsible for lower recruitment rates in these families compared to families with either normal-weight or obese children. Others described lack of time as the main reason for non-participation of families [8]. Consequently, researchers have often failed to reach the intended sample sizes [9], and have had to extend the recruitment periods [1], or modify inclusion criteria [10].

The mentioned problems mean it is important to evaluate effective recruitment strategies. These can be divided into active and passive strategies [11]. When using active strategies, researchers contact the target group of potential participants directly, e.g., through phone calls or targeted mailings. When using passive strategies, researchers spread information about their projects via electronic or print mass media or social networks. Potential participants identify themselves and are self-motivated to contact the research teams [11]. Inconsistent results were reported for the success of these strategies: active recruitment was more successful in an American pediatric intervention trial, whereas a passive strategy using media campaigns brought more eligible participants for a German study of obesity treatment for children [4, 12]. A pilot program to prevent weight gain in low-income mothers with overweight and obesity achieved best results when participants were directly recruited by trained staff [13]. Regarding direct recruitment, Gerards and colleagues [7] emphasized the need of special programs to train professionals in how to motivate families and how to increase their self-efficacy in dealing with the topic of obesity. In terms of cost effectiveness, direct mailing was the best way to recruit families in one obesity prevention trial [10]. Hare and colleagues found reactive approaches (mailing, media) more effective than proactive methods (referral through physicians) [14]. However, most of the recruitment strategies in the studies described above were applied in the context of clinical interventions and are per se not comparable with non-treatment studies. Though studies regarding recruitment strategies in observational prospective cohort studies are rare, there is some evidence that a combination of active and passive strategies via e.g., posters, online advertising, word of mouth (e.g., [15], investigating healthy pregnant women) and a centralized approach to participant recruitment [16] are effective. However, it remains unclear which recruitment methods are best suited for observational non-interventional family studies that include young children.

To shed some light on this topic, we examined which recruitment strategies were most successful in gaining participants for a longitudinal family study with the primary aim of investigating risk and protective factors for obesity in early childhood [17]. In the current study we investigated secondary outcomes of the larger study and aimed (a) to apply several active (e.g., face-to-face recruitment) and passive (e.g., advertisements on the internet, public transport, media) recruitment strategies, and to evaluate which were the most successful according to (b) the number of participants obtained and (c) cost effectiveness.

## Methods

### Study design and participants

This was part of a larger longitudinal family study, which was completed in 2014. The longitudinal study had been conducted within the Integrated Research and Treatment Center (IFB) AdiposityDiseases at the University Medical Center Leipzig. Inclusion criteria: Families with children aged 6 to 47 months. The risk group consisted of families with at least one parent with obesity (BMI ≥ 30) and the control group consisted of families with two parents of normal weight (BMI < 25). Exclusion criteria: Parents with overweight (25 ≤ BMI < 30). During the first phone contact families were asked how they had heard of the study. All analyses in this study are based on these information. In case of missing information we used further information participating parents gave in the study questionnaires.

### Process of recruitment

According to Walson, the process of recruitment consists of seven steps [18]. Below we will show how each step was implemented in this study.

#### Identifying eligible patient (in this case participant) population

This study took place in Leipzig. According to the official statistical yearbook 2010, Leipzig had 518,862 inhabitants, including 16,470 children aged from 6 to 47 months [19]. In Germany, the prevalence rates for obesity (BMI ≥ 30) in women of reproductive age range between 9.6% (18–29 years) and 17.9% (30–39 years) [20], yielding a mean score of about 13.8%. For men, the prevalence rate is 23.3% [20]. Hence, we estimated the likelihood of a child having a mother or father with obesity to be about 37% at the maximum. On this basis we estimated there were approximately 6090 children (aged 6 to 47 months) with at least one parent with obesity living in Leipzig. As assortative mating is a common phenomenon among obese individuals [21], we expected a much lower percentage of children with at least one parent with obesity (max. 3300). We also had to consider that some of the children aged between 6 and 47 months were siblings, which again reduced the pool of potential participants in this study.

#### Explaining the study

**Fig. 1:**
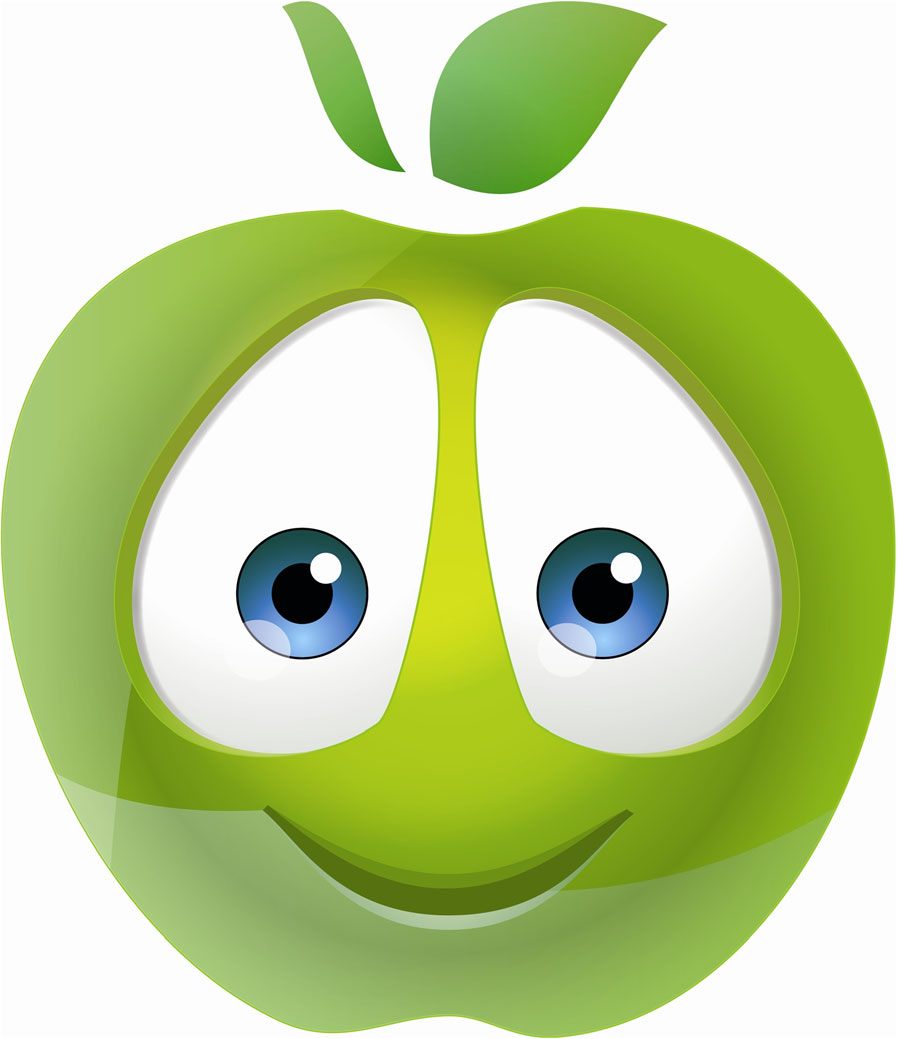
Corporate Design. The smiling green apple became the repeated motif of the family study with parents with obesity (Copyright © 2011 by Romy Schneider)

Before promoting the study, we created a unique campaign to motivate parents to participate. A smiling, green apple became the motif (Fig. 1). It was combined with the logo of the University Medical Center and the IFB AdiposityDiseases as a corporate design. As a part of public relations work we used a slogan (“Since when has chocolate been green? The way children eat”) to draw attention to the topic of child feeding and eating practices. Key information about the study was given as printed material. We asked normal weight parents and parents with obesity who had toddlers aged from 6 to 47 months to participate. The families were promised an expense allowance (€25) and a gift for the child.

#### Obtaining true informed consent

After parents had indicated their interest in participating, they received further comprehensive information by phone. Additionally, a letter was sent giving detailed information about the longitudinal design, the measures, the data needed about the child and the parents (e.g., weight development), and the families’ rights.

#### Maintaining ethical standards

The study was approved by the ethics committee of the Medical Faculty, University of Leipzig (case number 039–09/09032009). The families were repeatedly given information on their rights at every assessment point. They always had the right to withdraw from participation or to ask for data to be deleted.

#### Recruiting an adequate, representative sample

During the first 6 months of recruitment two problems became obvious: difficulties in, first, recruiting a sufficient number of obese participants with toddlers and second, recruiting a suitable comparison group that did not differ from the risk group in terms of education. This made it necessary to motivate non-academic families in particular to participate in the control group. The importance of promoting the study thus increased. Therefore, after 6 months of recruitment, we decided to extend the recruitment phase from 12 to 19 months for all established strategies. All recruitment strategies contained the same information about the incentives promised to the families. The sample yielded by this study was representative (in terms of education) for the area where we recruited, but generalization to other cultures may be limited. For recruitment we used the following strategies (subcategories of the main strategies are italicized).

##### Media

We worked closely with the departments of public relations of the IFB AdiposityDiseases and of the University Hospital. This enabled us to react to media inquiries in a timely fashion. Journalists from local newspapers and magazines wrote about the family study and interviewed the research team. Study information was given on local and national *radio* reports. Additionally, we inserted advertisements in local *newspapers* several times, and we used the *internet* for notifications (presentation of the study on various websites, advertisements and small ads).

##### Kindergartens

At first we tried to contact local kindergartens by *e-mail*. As we did not receive many responses and inquiries by families, we changed the strategy and got in touch by *phone and personal visits*. In this way, we contacted 80 of around 200 kindergartens in the city. We explained the study to the kindergarten teachers and asked them to hand the information material together with a small gift (sticker with a smiling apple, see Fig. 1) to the families. Altogether, 78% of the kindergarten teachers contacted assured us of their support. We offered the kindergartens a special conference for the parents, including a lecture on feeding/eating and feeding/eating problems where we would respond to parents’ questions.

##### Health professionals

We informed local *practitioners* (pediatricians, gynecologists, psychotherapists, midwives, physical therapists) of this study by phone calls, letters or visits, and asked them to display information material in their waiting rooms. Other researchers, for instance *members of the IFB AdiposityDiseases Outpatient clinic*, were asked for their support. Equally, all members of the Department of Child and Adolescent Psychiatry, Psychotherapy and Psychosomatics *(Clinic)* were informed.

##### Community-focused strategies

*Flyers* and *posters* were distributed to local supermarkets, children’s stores, playgrounds, and various public places such as clinics. For 2 weeks a poster was displayed on *public transport* (trams). We also focused on *face-to-face recruitment*: the research team was on the spot in *city/shopping centers,* at *public festivals* and in *clinics*. We talked to parents and handed out flyers to them.

All recruitment strategies yielded to costs of €56.25 per family on average (see results section) which was derived from the salaries of members of the research team who spent their working hours recruiting families or costs for printing materials (e.g., flyers, posters). In some cases participating families spread the information about our study to their friends and acquaintances who then contacted us. Hence, for these additional participants we incurred no direct costs (0 €).

#### Retaining subjects until study completion

So that families would keep us in mind, we sent Christmas cards and study reports with little gifts. Furthermore, we offered yoga classes for children. During the classes parents were able to follow a special open lecture on feeding/eating practices and feeding/eating problems.

#### Minimizing risk (in this case cost/benefit ratio)

We analyzed the success of the strategies used parallel to recruitment by asking every family during the first phone contact how they had heard of the study, which ensured that costs were minimized.

## Statistical analysis

We determined the number of families recruited through each strategy (main strategies and subcategories), distinguishing between risk group, control group and ineligible families. In line with Nguyen and colleagues, we summarized the costs (material expenses, staff appropriations, fees) of each recruitment path for a comparative cost analysis [3]. To conduct relative cost effectiveness analyses, we first calculated the mean cost per enrolled family over all recruitment strategies and for each strategy separately. We then used the calculated overall median to split the strategies into lower and higher cost strategies depending on whether the relative costs per family lay below or above the overall median costs. Finally, we divided the ranking of relative effectiveness into lower, middle or upper thirds according to the enrolment yield based on the total number of inquiries by families (for an analogous analysis see Nguyen and colleagues [3]). As the attrition rate in a longitudinal study can be considered as another indicator of the success of a recruitment strategy we calculated attrition rates for each strategy. We also tested whether the recruitment strategies differed regarding the attrition in the longitudinal study 11 months later by applying a Chi-square test for equality of distributions.

## Results

**Fig. 2:**
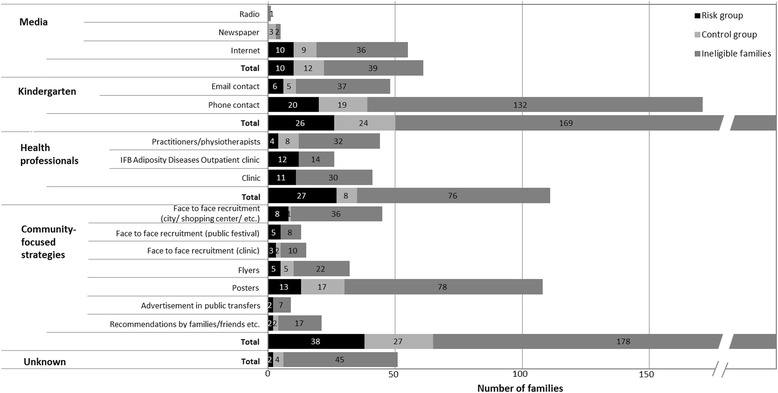
Numbers of enrolled and ineligible families for main recruitment. Strategies and subcategories

A total of 685 families contacted us following the different recruitment approaches. At the first phone contact, families gave information on the age of the children and on the weight and height of the family members. A large number of families were not eligible, mostly due to the BMI requirements (e.g., one or both parents was neither of normal weight nor obese) which were reported by parents on the phone, or they decided against participating after receiving detailed study information. However, 30% of the families who contacted us met the inclusion criteria and were willing to participate in the longitudinal study. This finally resulted in 209 families who participated from March 2011 to October 2012 (t1) However, of these families 31 families did not meet the BMI requirements after height and weight of the parents were assessed in the laboratory. Hence, a final sample of *n* = 178 families at t1 met the requested criteria. Overall, four main recruitment strategies and 15 subcategories were applied. Figure 2 shows the number of enrolled and ineligible families for each strategy. Altogether, of the four main strategies, the community-focused strategy was the most successful one, accounting for 36.5% of the sample (*n* = 65). This strategy was followed by contacts with kindergartens, which led to 28.1% of the sample (*n* = 50), and the help of health professionals, which yielded 19.7% of the sample (*n* = 35). Recruitment strategies using different media only accounted for 12.4% (*n* = 22) of the sample. When regarding the subcategories, recruiting families via contacting kindergartens by phone and recruiting families via posters were the most successful strategies yielding 21.9% (*n* = 39) and 16.9% (*n* = 30) of the sample, respectively. Furthermore, although 13.5% of fathers (*n* = 24) mentioned that their partner gave them the information and asked them to participate, no mother said she had been informed or motivated by her partner.

The estimated recruitment costs (hourly rates of the staff included) varied from €0.00 (community-focused strategies: recommendations) and €2.63 (media: internet) to €640 (community-focused strategies: face-to-face recruitment: clinic) per family. Table 1 shows the effectiveness of strategies according to a relative cost (higher and lower cost split at the median) and enrolment yield. As can be seen one of the most successful strategies was the recruitment via kindergartens using phone contacts. This strategy was one of the higher cost strategies, but with costs of €24.34 only slightly above the median. Of similar cost (€23.33) and also successful was the community-focused recruitment via posters which belonged to the lower cost strategies. Distributing flyers, face-to-face recruitment in clinics, at the city center/shopping center and public festivals as well as advertising in trams and via kindergartens using email contact was less effective and more expensive in this study.

**Table 1:** Relative effectiveness of recruitment strategies according to cost (per participant) and enrolment yields

	Lower cost^a^	Higher cost^a^
Upper third^b^	Community-focused strategies: ▪Posters	Kindergarten: ▪Phone contacts
Middle third^b^	Media: ▪Internet	
Lower third^b^	Media: ▪Newspapers, Radio Community-focused strategies: ▪Recommendations Health professionals	Community-focused strategies: ▪Flyers ▪Advertisement on public transport ▪Face-to-face recruitment at public festivals, at clinic, city center/shopping center Kindergarten: ▪Email contacts

^a^Median cost of €23.33 divides the strategies into lower and higher cost portions based on whether the estimated cost was below/equal or above the median

^b^Ranking of relative effectiveness is divided into upper (3.80–5.70% enrolled families), middle (1.90–3.79% enrolled families), and lower (<1.89 enrolled families) thirds. ▪/•Subcategories are named if there were differences in cost and relative effectiveness in a main strategy

Attrition rates from t1 to t2 in the longitudinal study ranged from 5.7% for recruitment via health professionals, 12.0% via kindergartens and 16.9% via community-based strategies to 18.2% for recruitment via media. There was no significant difference between the four main recruitment strategies regarding the attrition rates, *χ* ^2^(3) = 6.85, *p* = .08.

## Discussion

Recruiting participants with obesity for clinical trials is considered to be real challenge [5, 7, 8]. We experienced a similar challenge when recruiting participants for a non-interventional family study, both when recruiting participants with obesity and also when trying to enroll a suitable control group of normal weight. Contrary to Svensson and colleagues, who reported enhanced participation of families with low education and income levels [22], we had problems finding non-academic participants for the control group. The fact that low levels of education and income are associated with obesity in adults [23] and with a decreased willingness to participate in clinical research in general (e.g., [24]) might explain the difficulties described above, at least in part.

This resulted in an extended recruitment phase. The flexibility in the duration of the recruitment phase and the high number of strategies finally produced a sufficient sample size.

Finne and colleagues reached the majority of obese children (aged 8–16 years) in their study through passive strategies, especially via reports on TV and in newspapers [4]. In contrast, the most successful strategy in this study targeting families with young children was an active one. By indirectly contacting the families through spreading information material via kindergarten teachers we achieved a high level of recruitment. Moreover, for the targeted population in this non-interventional study, direct communication and explanations of the study to kindergarten teachers were essential to increase the willingness of families to take part. Although this type of contact (e.g., talks in kindergartens) was very time consuming and consequently very costly, it enhanced the willingness to participate. Robinson and colleagues reported that direct mailing (via mailing lists obtained from various databases) was the best strategy for recruiting families with children aged 4 to 7 years (BMI percentile ≥75, television consumption ≥14 h/week) for an obesity prevention trial aiming to reduce the time spent watching television [10]. We did not have access to the addresses of families in Leipzig. Thus, we tried emailing to kindergartens but we found we were much more effective when we switched to making phone calls. Emailing might be too impersonal and more likely to be ignored in this study. Phoning kindergarten teachers, explaining the study content to them and asking them to distribute the information material to the families yielded the most successful enrolment yield and was of moderate cost compared to other ways of recruitment in this study. Apparently, obesity and studies with toddlers are sensitive topics and parents need a sense of the trustworthiness of the researchers. Thus, parents’ positive reactions to recommendations from kindergarten teachers were probably based on their trust in these institutions and persons. However, following feedback from the professionals we agree with Gerards and colleagues that training in communication strategies and interview techniques may help people to talk about the topic of obesity [7].

Moreover, attracting attention to the study content using posters in locations as supermarkets, children’s stores, playgrounds, clinics and other public places was another successful way of recruiting families for our study. In terms of effectiveness it was even the most effective strategy (lower cost, high enrolment yield). In this study, the posters as well as other print material (flyers, stickers) carried a repeated motif to attract the attention of parents and children. During the assessment, staff received positive feedback by the families on the corporate design. In view of the success of recruitment via posters in this study, it might be useful for future studies similar to this one to consider devising campaigns that are suited to children and grab their attention and that of their parents. Also, in terms of using different types of media for recruiting participants, we recommend a presentation on the internet which was moderately effective (lower cost, moderate enrolment yield) in this study. However, it should be kept in mind that for other target groups in other populations and for other types of studies (e.g., interventional studies), different methods of recruitment from those presented in this study could also be efficient.

In this study, mothers informed fathers about the content of the family study and encouraged them to participate. One reason might be that mothers are closer to their toddlers (e.g., because they more often take parental leave) or are easier to motivate to take part in family studies. This should be considered when developing recruitment strategies. Since mothers are the ones who need to be targeted, media campaigns should be specifically tailored to them.

There are several limitations of the current study which deserve consideration. First, despite trying to obtain a population representative sample (e.g., via kindergartens and community-focused strategies), the sample in this study consisted of disproportionately motivated families in both groups. Second, some families might have been exposed to multiple recruitment strategies, e.g., they might have come into contact with print material on one occasion and with staff on another, while on the phone they probably reported only the most recent contact. Thus, for this subgroup of families in the investigated sample, it was not possible to determine which recruitment strategy led to the decision to participate. Third, we cannot rule out that the expense allowance and the gift for the child promised to the families in case of participation have altered the success rates of the recruitment strategies applied. For example, the recruitment methods used in this study might show different success rates when being used in a clinical study where participants receive an intervention.

## Conclusions

According to the results of the current study, only the combination of different routes and approaches led to the recruitment of a sufficient sample size of parents with obesity and parents with normal weight and their young children for a non-interventional family study. There were two outstanding strategies: Posters (community-focused strategies), and recruitment via kindergartens using phone contacts rather than emailing. These strategies produced the highest numbers of participants (high enrolment yield) at moderate cost. Expensive methods of recruitment such as advertising on public transport or face-to-face recruitment at various places have turned out not to be efficient for recruiting families with young children for a non-interventional study.
